# Duration of SARS-CoV-2 RNA detection in COVID-19 patients in home isolation, Rhineland-Palatinate, Germany, 2020 – an interval-censored survival analysis

**DOI:** 10.2807/1560-7917.ES.2020.25.30.2001292

**Published:** 2020-07-30

**Authors:** Sarah Omar, Christoph Bartz, Sabine Becker, Silke Basenach, Sandra Pfeifer, Corinna Trapp, Hildegard Hamm, Hans Christoph Schlichting, Magdalena Friederichs, Ulrich Koch, Christian Jestrabek, Ernst Hilger, Manfred Vogt, Klaus Jahn, Simiao Chen, Till Bärnighausen, Philipp Zanger, on behalf of the Palatina Public Health Study Group

**Affiliations:** 1 District Public Health Authority, Montabaur, Germany; 2 District Public Health Authority, Trier, Germany; 3 District Public Health Authority, Neustadt, Germany; 4 District Health Authority, Neuwied, Germany; 5 District Public Health Authority, Bad Ems, Germany; 6 District Public Health Authority, Wittlich, Germany; 7 District Public Health Authority, Kirchheimbolanden, Germany; 8 District Public Health Authority, Pirmasens, Germany; 9 District Public Health Authority, Germersheim, Germany; 10 District Public Health Authority, Cochem, Germany; 11 Federal State Agency for Consumer and Health Protection Rhineland-Palatinate, Koblenz, Germany; 12 Ministry of Health, Federal State of Rhineland-Palatinate, Mainz, Germany; 13 Heidelberg Institute of Global Heath, University Hospitals, Heidelberg, Germany; 14 Chinese Academy of Medical Sciences and Peking Union Medical College, Beijing, China; 15 Harvard Center for Population and Development Studies, Harvard University, Cambridge, United States; 16 Department of Global Health and Population, Harvard School of Public Health, Boston, United States; 17 Department of Infectious Diseases, Medical Microbiology and Hygiene, University Hospitals, Heidelberg, Germany; 18 The participating members of the Palatina Public Health Study Group are acknowledged at the end of the article

**Keywords:** COVID-19 diagnostic testing, patient isolation, survival analysis, review literature as topic

## Abstract

We analysed consecutive RT-qPCR results of 537 symptomatic coronavirus disease (COVID-19) patients in home quarantine. Respectively 2, 3, and 4 weeks after symptom onset, 50%, 25% and 10% of patients had detectable RNA from severe acute respiratory syndrome coronavirus-2 (SARS-CoV-2). In patients with mild COVID-19, RNA detection is likely to outlast currently known periods of infectiousness by far and fixed time periods seem more appropriate in determining the length of home isolation than laboratory-based approaches.

Most cases of coronavirus disease (COVID-19) are mild [1], making home isolation the standard containment measure for 80% of patients. Guidance on discontinuation of containment measures is hampered by incomplete knowledge on (i) the level and duration of detecting viral material in different patient groups, (ii) the infectious dose and (iii) the role of RT-qPCR as a proxy measure of infectiousness [2]. This uncertainty explains a significant heterogeneity in the current national recommendations on RNA laboratory testing for severe acute respiratory syndrome coronavirus-2 (SARS-CoV-2) as a tool for discontinuing isolation among countries in the European Union and European Economic Area (EU/EEA) [2]. 

Here, we present a review of currently published literature on the duration of SARS-CoV-2 RNA detection by RT-qPCR in the respiratory tract in various settings and patients groups. We analyse data of consecutive RT-qPCR results in 537 symptomatic patients with mild COVID-19 in home quarantine, collected from 28 February to 6 June 2020 in Rhineland-Palatinate, Germany. With this research, we aim to close a gap in current knowledge on the duration of RNA detection in patients with mild COVID-19 and inform test-based policy of discontinuing home isolation. 

## Literature review

We searched the PubMed online database on 8 April using the terms ‘SARS-CoV-2’ AND ‘shedding’ ‘NOT faeces’ and identified 85 results. Reviewing these papers and their reference lists yielded 11 studies that had analysed sequential RT-qPCR results in populations of 30 or more COVID-19 patients. One of these 11 studies did not provide any summary statistics on temporality [3] and another had been specifically selected for RNA detection of 30 days or longer [4], leaving nine studies [5-13] in populations of 32–146 COVID-19 patients for detailed comparison of populations, methods and results (Table 1).

**Table 1:** Reports providing descriptive statistics on duration of SARS-CoV-2 RNA positivity by RT-qPCR with at least 30 subjects and entry in PubMed by 8 April 2020 (n = 9)

Ref	Country	Setting	Severity	Population size	Detection period	Sampling interval^a^	Distribution of outcome in population (days)			Time to event analysis	Antivirals, comments
							Median or mean	IQR or SD	Maximum		
[5]	China	Hospital	53% moderate, 39% severe, 8% critical	137	Symptom onset to two negative results	Regular; every other day	Median: 20.0	IQR: 17.0–24.0	37	None	n = 41 lopinavir/ritonavir; analysis restricted to 137/191 survivors opens potential for selection bias
[7]	China	Hospital	Mild to moderate	56	Symptom onset to first negative result	Irregular	Median: 24.0	IQR: 18.0–31.0	42	None	None
[6]	China	Hospital	94% moderate, 6% severe	70	Symptom onset to first negative result	Irregular	Median: 22.0	IQR: 19.0–32.0	No data	None	Focus of study is on re-occurring positives
[8]	China	Hospital	74% moderate, 25% severe, 1% critical	120	Symptom onset to two negative results	Regular; every other day	Median_overall_: 23.0	IQR: 18.0–32.0	No data	Kaplan–Meier, Cox-regression	n = 78 lopinavir/ ritonavir and n = 42 no treatment
							Median_antiviral_: 22.0	IQR: 18.0–29.0			
							Median_non-antiviral_: 28.5	IQR: 19.5–38.0			
[9]	China	Hospital	75% non-ICU 25% ICU	32	Symptom onset to first negative result	Irregular	Mean_non-ICU_: 15.67	SD: 6.68	No data	None	None
							Mean_ICU_: 22.25	SD: 3.62			
[10]	China	Hospital	28% severe	113	Symptom onset to first negative result	Regular, daily	Median: 17.0	IQR: 13.0–22.0	No data	Kaplan–Meier	n = 55 umifenovir, n = 19 ribavirin
[11]	China	Hospital	83% non- severe; 17% severe	59	From first positive to first negative result	Regular, daily	Median: 14.0	IQR: 10.0–18.0	25	Kaplan–Meier, Cox-regression	All patients ribavirin; variation in definition of detection period may explain lower estimates
[12]	China	Hospital	86% moderate; 14% severe	147	Symptom onset to first negative result	Regular, every other day	Median: 17.0	IQR: 12.0–21.0	47	Kaplan–Meier	All patients received antiviral treatment, ‘most commonly ribavirin and interferon’
[13]	Korea	Outpatient	100% mild	199	Diagnosis to two negative results	Irregular; every other day to once a week	Mean_overall_: 24.5	SD: 4.8	No data	none	53 asymptomatic and 146 symptomatic subjects
							Mean_symptomatic_: 25.2	SD: 4.9			
							Mean_asymptomatic_: 22.6	SD: 4.0			

ICU: intensive care unit; IQR: interquartile range; Ref: reference; SD: standard deviation.

^a^ Interval of sampling for RT-qPCR.

All nine studies were conducted in East-Asia (eight in China, one in South Korea) and most of them in a hospital setting (n = 8). Substantial proportions of the included patients suffered from severe COVID-19 and received antiviral medication [5-13]. The methodology and reported estimates were heterogeneous. All but two studies [11,13] used the period from symptom onset to time of first negative RT-qPCR result or time to two consecutive negative RT-qPCR results as outcome of interest. Only half of the studies sampled on a regular basis; daily (n = 2) or every other day (n = 3). Most research, with the exception of one study [13], seemed to use data from irregular (i.e. convenience) sampling. Four studies used survival analysis methods [8,10-12] but none acknowledged the nature of the data by using interval-censoring [14,15]. This increases the risk of systematic bias and overestimation, particularly in those studies with irregular sampling intervals [6,7,9]. In the seven studies that reported the median duration of RNA detection, the estimates varied from 14 to 24 days. In the remaining two studies, one group reported a mean estimate of 24.5 days. The other study found means of 22.25 and 15.67 days for the intensive care unit (ICU) and non-ICU patient sub-population, respectively. Only one of the nine reviewed studies had looked at COVID-19 patients with mild disease in an outpatient setting; it found viral RNA to be detectable in symptomatic patients for a mean duration of 25.2 days. All nine studies on duration of RNA detection studied small samples, explaining wide measures of spread around the reported estimates. As far as we are aware, there are currently no published data on the duration of RNA positivity in the upper respiratory of patients with mild COVID-19 that could inform a public health assessment of RT-qPCR as a tool for monitoring home isolation.

## Retrospective study using routine data from district public health authorities

Rhineland-Palatinate is one of 16 federal states in Germany and has a population of about 4 million people. Containment of mild COVID-19 cases is managed by 24 district public health authorities (DPHA) responsible for 35 districts. All 24 DPHA received an invitation to retrospectively enter into a EUSurvey database (https://ec.europa.eu/eusurvey/home/welcome) information on demographic characteristics and consecutive RT-qPCR results (‘positive’, ‘negative’ or ‘equivocal’) of COVID-19 patients who were in home isolation. No personal identifying information was stored in the online database.

A person was included in the analysis (i) if they were a laboratory-confirmed, RT-qPCR-positive case of COVID-19, (ii) if they were in home isolation between 28 February 2020 (date of first notified COVID-19 case in Rhineland-Palatinate) and 6 June 2020 (end of study period), (iii) if they had tested negative in RT-qPCR in at least one upper respiratory tract sample provided after diagnosis and before 6 June 2020, and (iv) if they had symptoms of COVID-19 before the first (i.e. diagnostic) RT-qPCR test.

For observations with missing information (n = 98), we imputed the date of symptom onset by subtracting the average delay of notification of 5.56, rounded to 6 full days. The latter was calculated from all statutory notifications made by 6 June 2020 that had data on both date of onset and date of statutory notification.

Data were extracted from the online platform on 6 June 2020, imported into Stata, cleaned, and RT-qPCR-results were binarised by recoding ‘equivocal’ to ‘positive’. We used interval-censored survival analysis (‘stintreg’ command, Stata 15) to estimate survival time, with the left margin of the censoring interval being days between date of onset and last positive swab and the right margin being days between date of onset and first negative swab. Time ratios comparing the median time to RNA negativity in the exposed with that in the unexposed were calculated for several exposure variables. Goodness of fit of different parametric survival models to our data was assessed using the Akaike information criterion [16] and visually by plotting the cumulative hazard function [17] against the Cox–Snell residuals.

Since RT-qPCR analyses were provided by different microbiology laboratories, information on type of RT-qPCR assay, cycle threshold (CT) or raw data on individual CT values were not available. To account for the additional level of variation introduced by the use of different RT-qPCR assays in different microbiology laboratories, we adjusted all regression analyses for clustering by DPHA.

## Ethical statement

All data presented were collected in response to the SARS-CoV-2 pandemic and in accordance with the German Infection Protection Act. Institutional review and individual informed consent was not sought.

## Duration of RNA positivity

Sixteen of 24 district public health authorities in Rhineland-Palatinate submitted data for overall 603 patients. On further review, 53 of them were asymptomatic and 13 had missing information on symptoms, leaving 537 symptomatic COVID-19 patients in home isolation for further analysis (Table 2).

**Table 2:** Baseline characteristics of symptomatic COVID-19 patients, Rhineland-Palatinate, Germany, 28 February–6 June 2020 (n = 537)

Exposure characteristic			
	**n**	**%**	
Male	233	43.4	
Female	304	56.6	
Date of onset unknown^a^	98	18.2	
At least one ‘equivocal’ laboratory result recoded as ‘positive’	17	3.2	
Immunosuppression^b^	7	1.3	
Treatment before home isolation			
Inpatient	67	12.5	
Outpatient	425	79.1	
Unknown	45	8.4	
Epidemiological context at time of diagnosis			
Healthcare staff	81	15.1	
Patient in hospital	19	3.5	
Nursing home resident	18	3.4	
Community^c^	398	74.1	
Unknown	21	3.9	
	**n**	**Median**	**IQR**
Age in years (in five equally sized groups)			
1st	114	26.0	21.0–29.0
2nd	110	38.0	35.0–41.0
3rd	108	49.5	46.0–51.5
4th	105	57.0	55.0–60.0
5th	100	71.0	64.0–81.0
RT-qPCR			
Median number of tests per subject until first negative test	537	2.0^d^	2.0–3.0
Median time to first negative test in days	537	20.0^e^	16.0–28.0
Length of censoring intervals^f^ in days	537	10.0^g^	7.0-15.0

IQR: interquartile range.

^a^ Imputed by date of notification minus delay of notification of 6 days.

^b^ Pregnancy (n = 2), renal transplant and dialysis (n = 2), cancer (n = 2), rheumatoid arthritis (n = 1).

^c^ Not belonging to an institutional transmission context or cluster.

^d^ Minimum–maximum: 2–9 tests.

^e^ Minimum–maximum: 1–68 days.

^f^ Defined as time from last positive to first negative test result in days.

^g^ Minimum–maximum: 1–68 days.

**Figure 1:**
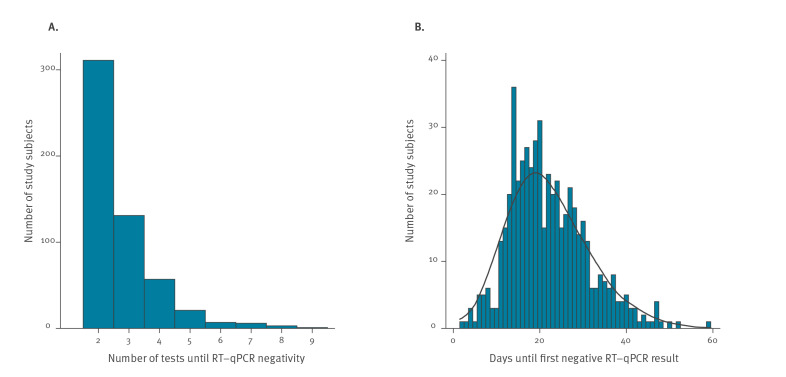
Number of samples analysed per subject and days until first PCR-negative swab in home-isolated COVID-19 patients, Rhineland-Palatinate, Germany, 2020 (n = 537)

**Figure 2:**
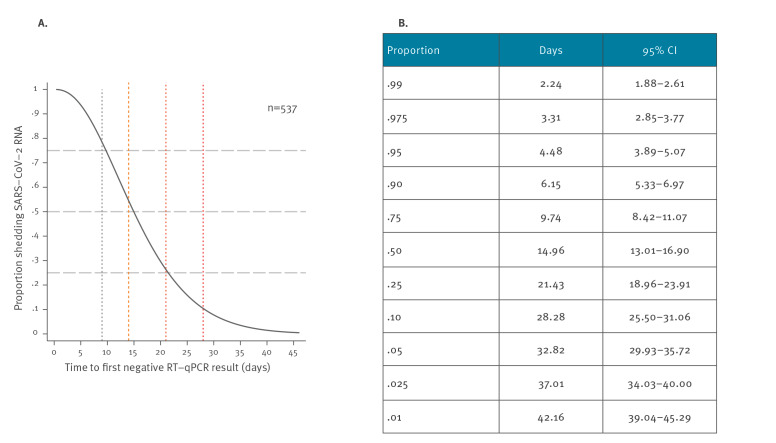
Duration of SARS-CoV-2-RNA positivity in COVID-19 patients in home isolation, Rhineland-Palatinate, Germany, 2020 (n = 537)

CI: confidence interval; SARS-CoV-2: severe acute respiratory syndrome coronavirus-2.

Figure 1 displays key statistics of the number of RT-qPCR tests and time periods analysed for this study. A generalised gamma distribution fit our data best (Supplementary Table S1 and Supplementary Figure). We found that respectively 50%, 25%, and 10% of patients were still positive for SARS-CoV-2 RNA 2, 3 and 4 weeks after the onset of symptoms. At 14 days after onset, the earliest moment to discontinue home isolation currently recommended in Germany [18], 53.5% of COVID-19 patients still had detectable SARS-CoV-2 RNA (Figure 2). Repeating the analysis after exclusion of 17 patients with ‘equivocal’ RT-qPCR results recoded as ‘positive’ changed our results only marginally: we found between 0.33 and 1.00% shorter time to SARS-CoV-2 negativity estimates than those displayed in Figure 2B. Comparing the results of the interval-censored analysis (Figure 2) with the distribution of time periods until the first negative test result (Figure 1B) shows that interval censoring gave a ca 5 days shorter estimate for the median RNA detection time (14.96 versus 20 days).

A. Number of samples for RT-qPCR from respiratory material taken until first negative result. More than half of the patients were already negative for SARS-CoV-2-RNA at the time of the second test (percentile_0.50_ (P_0.50_) = 2 tests) and 75% at the third PCR-test (interquartile range: 2–3 tests). The remaining quarter of the study population needed up to nine tests to become RT-qPCR-negative (P_0.85_ = 4; P_0.95_ = 5; P_0.99_ = 7; maximum = 9 tests).

B. Days from onset of symptoms until first SARS-CoV-2 RT-qPCR-negative swab from respiratory material. Grey line: kernel density plot (Epanechnikov). Half of the population tested negative on the 20th day (interquartile range: day 16–28) after onset of symptoms; the earliest negative test was on the second and the latest negative test on the 68th day after onset of symptoms; one subject with first negative test result beyond 60 days was excluded for more condensed display of distribution.

Cumulative proportion of SARS-CoV-2 RNA detection in upper respiratory tract samples at various time points after onset of symptoms; estimates from interval-censored survival analysis based on data from 537 symptomatic COVID-19 patients and univariable generalised gamma accelerated failure time regression model.

A. Cumulative proportion of population detection SARS-CoV-2 RNA as a function of time onset of symptoms. Horizontal dashed reference lines at 75, 50 and 25 population percentiles; vertical dotted reference lines at 9 days (grey), 14 days (yellow, i.e. minimum length of home isolation in Germany by 13 June 2020 [18]), 21 days (orange) and 28 days (red). Current best estimate of duration of infectiousness: 8 days [3,19,20].

B. Estimates of ‘days after symptom onset’ with 95% CI for detection of SARS-CoV-2 RNA in cumulative population proportions; Proportion = proportion of population positive for SARS-CoV RNA.

In a model adjusting for all exposure characteristics at once, patients in the oldest age group had 14% shorter median time to PCR negativity than those in the youngest age group (time ratio: 0.86, p = 0.037), while no such differences were found for the remaining age groups. Similarly, nursing home residents had 28% shorter median time to RNA negativity than patients with no specific epidemiological context at time of diagnosis (time ratio: 0.72; p = 0.013). Hospitalisation before home isolation, by contrast, was associated with 26% longer duration of RNA positivity compared with patients in home isolation throughout COVID-19 (time ratio: 1.26; p = 0.049) (Supplementary Table S2). Using a model adjusting for all exposure characteristics at once to predict time to RNA negativity for given population proportions only marginally changed the results from the unadjusted model (Figure 2B, Supplementary Table S3).

## Contextualisation of findings

While several countries recommend negative laboratory testing for particular patient groups before discontinuing isolation [2], there is increasing evidence that the period when viral nucleic acid can be detected may exceed the period of infectivity by far. Studying 77 infector–infectee transmission pairs, He at al. found convincing evidence for substantial pre-symptomatic transmission that peaks shortly before the onset of symptoms and then declines until Day 8 after symptom onset [3]. Support for an end of transmissibility around Day 8 after onset of symptoms comes from two independent studies that were not able to propagate virus in cell cultures from RT-qPCR-positive individuals beyond that point in time [19,20]. Against this background, and irrespective of the potential reason for this discrepancy (i.e. higher sensitivity of RT-qPCR or detection of non-replicative RNA remnants), our finding that 78% of patients in home isolation remained RT-qPCR-positive beyond Day 8 after onset of symptoms strongly supports that in patients with mild COVID-19, positive nucleic acid testing results do not allow any conclusions on infectivity. Similarly, respectively 50%, 25% and 10% of all patients would be kept overly long at home when choosing the end of the infectious period on Days 14, 21 and 28 (+/−2 days) after onset of symptoms and hereby cautiously taking into account evidence from one report on prolonged detection of replicative virus in one mild case of COVID-19 [21]. Therefore, RT-qPCR-guided containment leads to additional costs through prolonged periods of isolation and repeated sampling and increases the individual and social burden of the current epidemic.

 Compared with published estimates on duration of RNA detection after onset of symptoms, ranging from 14 to 24 days for the median and 15.67 to 24.5 days for the mean, our estimate of the mean of 14.96 days is at the lower end. It is difficult to tell whether this is a result of differences in study population or design, or both. From a methodological point of view, however, we expected that our analysis using interval-censoring (i.e. the interval between the last positive and the first negative swab) would reduce systematic information bias compared with estimates derived from time to first negative swab and would thus lead to shorter estimates of time to RNA negativity than most of the reviewed studies. Accordingly, we found that in our population and setting, the median duration of RNA detection using interval-censoring was ca 5 days shorter than the median time to the first negative test (14.96 versus 20 days).

## Conclusion

RT-qPCR testing seems to be of limited value in guiding the duration of home isolation in mild COVID-19. Negative follow-up testing may be useful in providing certainty about non-infectiousness before ending the isolation in settings where onward transmission has particularly detrimental consequences (e.g. healthcare setting) or in patient groups with known risk factors for prolonged viral shedding and the associated risk of infectiousness (e.g. the severely immunosuppressed). For cases where laboratory monitoring is indispensable, knowledge of the RT-qPCR threshold cycle may improve our judgement on whether a positive result indicates infectiousness or not [20]. By contrast, positive follow-up tests correlate poorly with infectiousness. Hence, after the diagnosis of COVID-19 is established, the large proportion of positive results that emerge from RT-qPCR monitoring during the first 4 weeks after onset of symptoms, and that exceed the period of infectiousness by far, do not meaningfully contribute to the anti-epidemic management of mild COVID-19 cases. Instead, they increase the overall cost and social burden of the epidemic without mitigating it. We conclude that for most patients with mild COVID-19, the use of fixed time periods, based on sound estimates of the infectious period, seem more appropriate in guiding the duration of containment measures than laboratory-based approaches. More research on risk groups and factors associated with prolonged infectiousness (e.g. immunosuppression, age and disease severity) is needed to tailor fixed time periods to various settings and groups and ultimately further reduce the use of RT-qPCR in guiding the duration of SARS-CoV-2 containment.

## Supplementary Data

186000 Supplement
