# Supplementary material for: Duration of SARS-CoV-2 RNA detection in COVID-19 patients in home isolation, Rhineland-Palatinate, Germany, 2020 – an interval-censored survival analysis
Source: Euro Surveill. 2020 Jul 30;25(30):2001292. doi: 10.2807/1560-7917.ES.2020.25.30.2001292 (PMC7393848; doi:10.2807/1560-7917.ES.2020.25.30.2001292)
Supplement: Supplement [file 20-01292_ZANGER_Supplement.pdf]

Material supplementary to

Omar et al.: **Duration of SARS-CoV-2-RNA shedding of COVID-19 patients in home-isolation, Rhineland-Palatinate, Germany, 2020 – an interval-censored survival analysis**

published in Eurosurveillance 2020.

This supplementary material is hosted by Eurosurveillance as supporting information alongside the article 'Duration of SARS-CoV-2-RNA shedding of COVID-19 patients in home-isolation, Rhineland-Palatinate, Germany, 2020 – an interval-censored survival analysis', on behalf of the authors, who remain responsible for the accuracy and appropriateness of the content. The same standards for ethics, copyright, attributions and permissions as for the article apply. Supplements are not edited by Eurosurveillance and the journal is not responsible for the maintenance of any links or email addresses provided therein.

Supplementary table 1: Comparison of different parametric survival-time models fitted to data on RNA-shedding of 537 COVID-19 patients in home-quarantine, Germany, 2020

Exposure characteristic	Time ratio*				
	G.-Gamma	Exponential	Weibull	Log-logistic	Log-normal
Age group					
1st (youngest, baseline)	1.00	1.00	1.00	1.00	1.00
2nd	0.9513482	0.9370667	0.9497597	0.9633262	0.9618845
3rd	0.9415026	0.9363331	0.9466682	0.9299457	0.9194536
4th	1.010456	0.9929827	1.026379	0.9617478	0.9658205
5th (oldest)	0.8620867	0.8574587	0.8626436	0.8545677	0.8515137
Male sex (baseline)	baseline	baseline	baseline	baseline	baseline
Female sex	1.069046	1.07409	1.062266	1.087074	1.09808
Epidemiological context at time of diagnosis					
none of the above (baseline)	1.00	1.00	1.00	1.00	1.00
health care staff	0.9967623	0.9939108	0.9961743	0.9904499	0.9849082
patient in hospital	0.7591267	0.7059759	0.8145882	0.5595564	0.5709811
nursing home resident	0.7191241	0.6889845	0.7282318	0.6973209	0.6925642
unknown	0.8573386	0.820859	0.8623858	0.8390834	0.8259922
Treatment before home-isolation					
outpatient (baseline)	1.00	1.00	1.00	1.00	1.00
inpatient	1.258227	1.277183	1.239303	1.338824	1.335933
unknown	1.088437	1.119524	1.060157	1.14811	1.156522
Immunosuppression					
no (baseline)	1.00	1.00	1.00	1.00	1.00
yes	1.023005	1.079835	0.9963424	1.080005	1.0955
Constant	16.01236	14.38278	17.31223	13.32915	12.82708
Ancillary	0.5267122		1.968469	0.3124015	0.5491062
Kappa	0.7270878				
Log likelihood	-697.5152	-807.66094	-698.9066	-711.8593	-706.8228
Akaike's Information Criterion	1425.230	1641.322	1425.813	1451.708	1441.646

Table displays results from multi-variable interval-censored accelerated failure time regression models

* ratio of median time to RNA-negativity among those exposed compared to those unexposed (baseline)

Supplementary figure. Goodness of fit plots

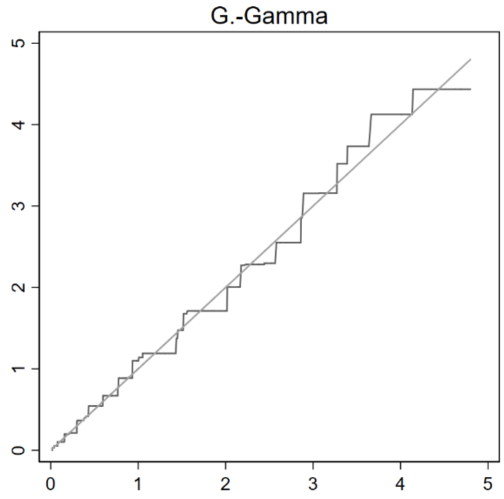

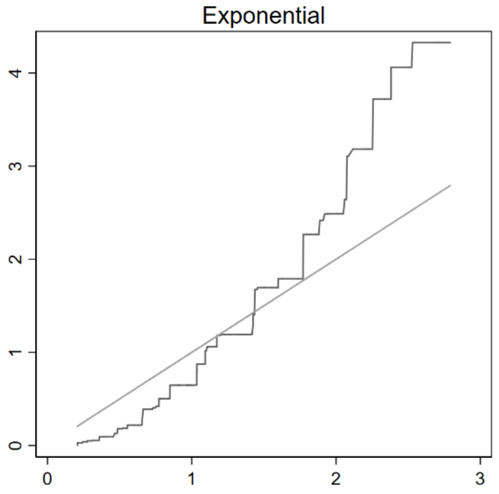

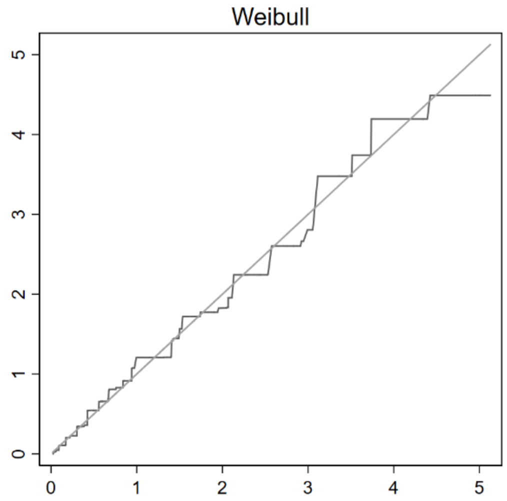

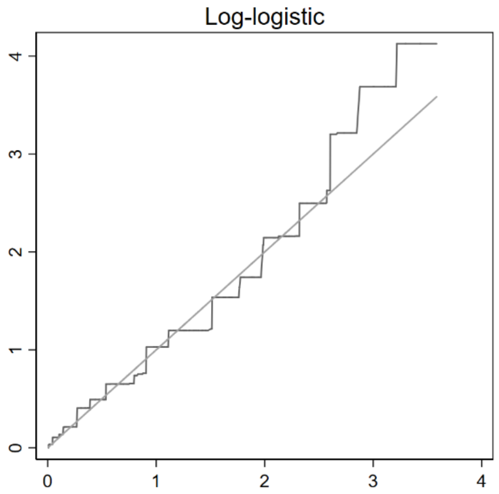

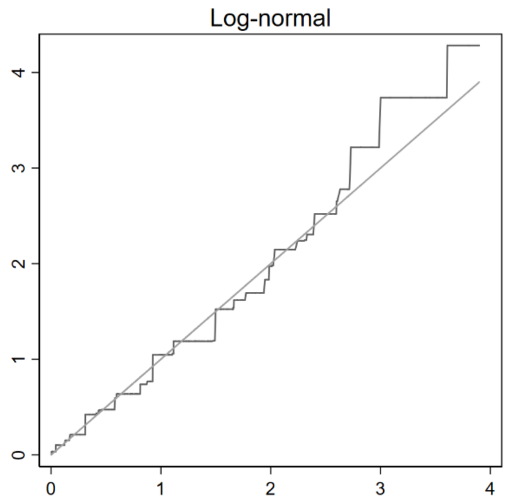

Graphs plot the cumulative hazard (y-axis) versus Cox-Snell residuals (x-axis) from different parametric models fitted to interval-censored data on RNA-shedding of 537 COVID-19 patients in home-isolation; generalized gamma distribution fitted the data best

Supplementary table 2: Association of baseline characteristics with time to RNA-negativity in 537 home-isolated COVID-19 patients, Germany, 2020

Exposure characteristic	n	Time ratio*	95% CI	Median time to RNA negativity (in days)	P
Age					
1st (youngest, baseline)	114	1.00	n.a.	15.58	n.a.
2nd	110	0.9513482	0.7826201 to 1.156453	14.80	0.617
3rd	108	0.9415026	0.8402835 to 1.054914	15.01	0.299
4th	105	1.010456	0.8571059 to 1.191243	15.95	0.901
5th (oldest)	100	0.8620867	0.749781 to 0.991214	13.63	0.037
Male sex (baseline)	233	baseline	n.a.		n.a.
Female sex	304	1.069046	0.962196 to 1.187763		0.214
Epidemiological context at time of diagnosis					
none of the above (baseline)	398	1.00	n.a.	15.26	n.a.
health care staff	81	0.9967623	0.917025 to 1.083433	15.36	0.939
patient in hospital	19	0.7591267	0.514994 to 1.11899	12.84	0.164
nursing home resident	18	0.7191241	0.554403 to 0.932786	11.59	0.013
unknown	21	0.8573386	0.568339 to 1.293295	14.00	0.463
Treatment before home-isolation					
outpatient (baseline)	425	1.00	n.a.	14.84	n.a.
inpatient	67	1.258227	1.00137 to 1.58097	15.85	0.049
unknown	45	1.088437	0.914305 to 1.29573	14.42	0.341
Immunosuppression					
no (baseline)	530	1.00	n.a.	14.94	n.a.
yes	7	1.023005	0.779921 to 1.34186	14.80	0.869

Table displays results from multi-variable interval-censored accelerated failure time regression

* association of independent variables expressed as ratio of median time to RNA-negativity among those exposed compared to those unexposed (baseline)

Supplementary table 3. Duration of SARS-CoV-2-RNA shedding in COVID-19 patients in home-isolation, adjusted for age, sex, epidemiological context, treatment setting, and immunosuppression, Germany, 2020

Proportion	Days	95% CI
.99	2.41	1.85 - 2.96
.975	3.49	2.98 - 3.99
.95	4.66	4.17 - 5.16
.90	6.32	5.69 - 6.97
.75	9.89	8.66 - 11.11
.50	15.01	13.02 -17.01
.25	21.37	18.85 - 23.88
.10	28.08	25.50 - 30.67
.05	32.53	30.07 - 35.00
.025	36.44	34.29 - 39.00
.01	41.71	39.24 - 44.17

Cumulative proportion of population shedding SARS-CoV-2 RNA from upper respiratory tract by various time-points after onset of symptoms; estimates from interval-censored survival analysis based on data on RNA-shedding from 537 symptomatic COVID-19 patients and multivariable generalized gamma accelerated failure time regression model. Proportion=proportion of population shedding SARS-CoV-RNA from upper respiratory tract; CI=confidence interval
